# Anthelmintic resistance of gastrointestinal nematodes in dairy calves within a pasture‐based production system of south West Western Australia

**DOI:** 10.1111/avj.13162

**Published:** 2022-04-05

**Authors:** M. Mauger, G. Kelly, C. H. Annandale, I. D. Robertson, F. K. Waichigo, J. W. Aleri

**Affiliations:** ^1^ School of Veterinary Medicine, College of Science, Health, Engineering and Education Murdoch University Murdoch Western Australia Australia; ^2^ Boehringer Ingelheim Animal Health Australia Pty. Ltd. North Ryde New South Wales Australia; ^3^ College of Veterinary Medicine Huazhong Agricultural University Wuhan Hubei China; ^4^ Brunswick Veterinary Services Brunswick Junction Western Australia Australia; ^5^ Centre for Animal Production and Health, Future Foods Institute Murdoch University Murdoch Western Australia Australia

**Keywords:** anthelmintics, dairy calves, doramectin, fenbendazole, levamisole, macrocyclic lactones

## Abstract

The objective of this study was to determine the prevalence of gastrointestinal nematodes among post‐weaned calves aged between 4 and 12 months old within a pasture‐based system of south west Australia and quantify the level of anthelmintic resistance. Pre‐treatment FECs were monitored on 14 dairy farms. Anthelmintic resistance was assessed on 11 of the farms. Control FECs were compared with anthelmintic FECs at 14 days post‐treatment with doramectin (injectable), levamisole (oral), fenbendazole (oral) and a levamisole/abamectin combination (pour‐on). Results demonstrate a strong level of anthelmintic resistance, with at least one class of anthelmintic failing to achieve a 95% reduction in FEC in one or more gastrointestinal nematode species. Doramectin was fully effective against *Ostertagia*, but *C. oncophora* displayed resistance in 91% of the farms. Conversely, levamisole was fully effective against *C. oncophora*, but *Ostertagia* displayed resistance in 80% of the farms. Fenbendazole resistance was present in both *C. onocphora* and *Ostertagia* in 64% and 70% of the farms, respectively. *Trichostrongylus* showed low resistance, occurring in doramectin (14%) and levamisole/abamectin combination (14%). This study confirms that anthelmintic resistance is common. Regular FEC reduction testing is recommended to monitor and guide decision‐making for appropriate anthelmintic usage.

T*richostrongylus axei, Ostertagia ostertagi* and *Cooperia oncophora* are the predominant and important gastrointestinal nematodes (GIN) of cattle.^1^ Among these, *O. ostertagia* is the most pathogenic species, characterised by severe burdens in the first season of grazing among calves, causing type I Ostertagiosis. This manifests with marked weight loss, profuse watery diarrhoea, inappetence and mortality.^2^, ^3^ Type II Ostertagiosis is related to a high emergence of inhibited larvae with clinical signs identical to type I disease in older calves and adult cattle, with a primary clinical sign of inappetence in less severely infected animals.^3^ This may manifest as acute outbreaks due to larval emergence or as chronic cases.^3^, ^4^ Despite being less harmful than *T. axei* and *O. ostertagi*, *Cooperia* species have been associated with production losses such as marked weight loss in young stock,^5^, ^6^ clinical disease in adult cattle and as parasitic gastroenteritis due to mixed infections with *O. ostertagi*.^2^

In cattle production systems, the major risk factors for GIN parasitism include parasite characteristics (fecundity, hypobiotic larvae, transmission, morphology), host factors (genetic resistance, physiological status, immune immunity) and environmental factors (nutrition, husbandry practices, management, climate).^7^, ^8^ First season grazing calves are at major risk as they are the most susceptible to clinical disease due to an underdeveloped immune system,^7^ especially when raised on permanent pastures.^5^ Gastrointestinal nematode species with increased fecundity and hypobotic capabilities that are able to survive unfavourable environmental and host conditions, allow accelerated infection rates and the successful dissemination of resistant alleles to subsequent GIN generations.^2^, ^9^ Animal husbandry and management practices that can result in an increase in anthelmintic resistance include early weaning onto a pasture‐based diet,^10^ failure to provide quarantine treatments in new stock^11^ and intensive anthelmintic treatment regimens.^12^, ^13^

Anthelmintic resistance is defined as being present when, ‘there is a greater frequency of individuals within a population that are able to tolerate doses of a compound than in a normal population of the same species and is heritable’.^14^ Several studies within New Zealand and Australia^15^, ^16^ have reported anthelmintic resistance, with similar results reported overseas including the United States,^17^ England^18^ and Argentina.^19^ In Australia, a recent study in the eastern states reported anthelmintic resistance in 20 commercial dairy farms among replacement heifers.^10^ Anthelmintic resistance was detected against doramectin, levamisole and fenbendazole anthelmintics.

There is limited information on the anthelmintic resistance profiles in the south west region of Western Australia dairy farms. Dairy farming in Western Australia is characterised by a predominantly pasture‐production‐based system under Mediterranean conditions characterised by hot summers and relatively mild winter temperatures.^20^ Data and information on anthelmintic resistance profiles will provide evidence‐based strategic management strategies of anthelmintics.^21^ The objective of this study was to determine the prevalence of GIN among post‐weaned replacement heifers and bull calves aged between 4 and 12 months old in dairy farms within a pasture‐based production system of south western Australia and to quantify the level of anthelmintic resistance.

## Materials and methods

### Study area and approval

The study was conducted in the south west region of Western Australia. The region has a temperate Mediterranean climate with an annual rainfall of approximately 730 mm. The study was conducted between June and December 2020 in accordance with the Australian Code of Practice for the Care and Use of Animals for Scientific Purposes. The study was approved by the Animal and Human Ethics Committees of Murdoch University (Approval No. R3213/20 and 2020/006, respectively).

### Study design and sampling

A convenience sample of 14 dairy calf herds was included in the study with a total of 1,271 calves. The selection criteria for inclusion into the study was calf availability, good animal identification methods, physical restraint facilities and willingness to participate in the study. On each farm, 75–100 post‐weaned replacement heifer and bull calves aged between 4 and 12 months old were enrolled into the study. A total of 11 farms were enrolled into the second part of the trial, which involved assessing for anthelmintic resistance based on FEC of ≥500 eggs per gram (epg) in at least 10%–15% of the samples. The secondary visit was conducted 10–14 days post‐anthelmintic treatment, and thereafter, faecal samples were analysed for FECs, larval culture and differentiation and anthelmintic resistance quantification.

A questionnaire template (Data S1) was used to capture farm data and anthelmintic management strategies such as pasture management, frequency, type and decisions on anthelmintic use.

### General data collection

A minimum of two farm visits were conducted for each farm sampled. The activities included collection of faecal samples, measurement of body weights using calibrated scales and allocation of the sampled individuals into four respective anthelmintic treatment groups and a control group. Faecal samples from the calves on the initial farm visit were used to determine individual FEC load, whereas faecal samples from the second visit were used to determine faecal egg count reduction (FECR), larval culture and the quantification of anthelmintic resistance. Briefly, faecal samples were collected directly from the rectum, calves weighed and assigned into the respective treatment groups. Samples were stored at 4°C until processing to prevent faecal worm eggs from further development and hatching. Samples from the initial visit were processed within four days maximum of collection. Secondary samples were overnight posted with cooling the following business day to the Department of Primary Industries and Regional Development to reduce duration outside a 4°C environment. Calves were weighed using an electronic cattle scale (W110 Livestock Weighing System, Gallagher Group Limited) prior to anthelmintic treatment and allocated into one of five treatment groups. These were (1) untreated controls; (2) ML (doramectin) 0.2 mg/kg SC (Dectomax®, Zoetis Australia); (3) BZ (fenbendazole) 7.5 mg/kg Oral (Panacur 100®, MSD Animal Health Australia); (4) LV (levamisole hydrochloride) 8 mg/kg Oral (Nilverm LV®, MSD Animal Health Australia) and (5) LV/ML (10 mg/kg levamisole, 0.5 mg/kg abamectin) 1 ml/20 kg (Eclipse Combination Pour‐on®, Boehringer Ingelheim). Treatment groups were evenly spilt depending on the number of calves included from each respective farm, with a minimum of 15 calves per group. Allocation of calves were randomised using a randomly generated treatment list for each respective farm based of the number of calves, where treatment was allocated dependent on the order calves were sampled during the initial visit.

### Laboratory analysis

#### FEC, larval differentiation and quantification of anthelmintic resistance

Initial FEC samples were examined using the Modified McMaster Technique.^22^ Slides were observed under a light microscope at ×10 magnification, and all faecal worm eggs recorded in eggs per gram. All FECs were performed by a single operator using 2 g of faeces in 60 ml of saturated sodium chloride (NaCl), where 2 chambers were counted and one egg equated to 50 epg. *Strongyle* and *Nematodirus* species were pooled into a single count for total initial farm FEC. Post‐anthelmintic treatment faecal samples were submitted to the Department of Primary Industries and Regional Development for larval cultures, differentiation and anthelmintic resistance quantification.

### Statistical data analysis

Data were analysed using SPSS Statistics software version 22.0, 2013 (SPSS Inc., Chicago 111). Descriptive statistics were generated, and FECR was calculated by comparing the post‐treatment arithmetic mean FECs, 100 $\left(1-\left[\bar{x}t/\bar{x}c\right]\right)$ where $\bar{x}\;$ is the mean, *t* is the treated group FEC and *c* is the control group.^23^ Anthelmintic resistance was defined as <95% reduction in FEC with a lower 95% confidence interval (CI) of <90%.^24^

## Results

### General descriptions

**Figure 1:**
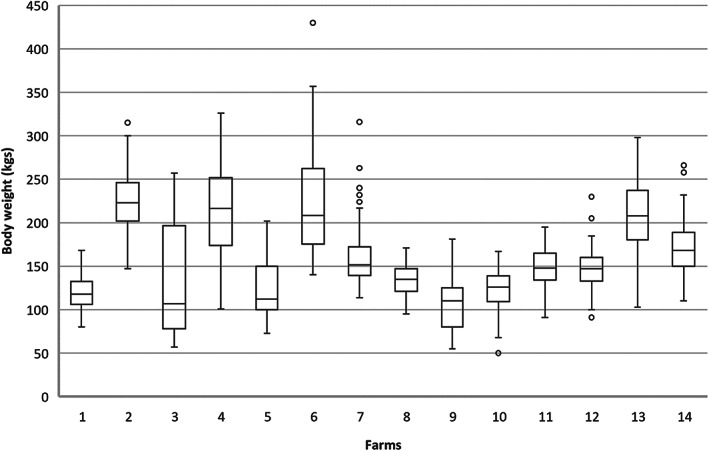
The distribution of median body weight for animals aged between 4 and 12 months, sampled across 14 dairy farms in the south west region of Western Australia between June – December 2020.

A total of 1,271 calves from 14 dairy herds were sampled. The median age of animals sampled was 6 months (range, 4–11 months). A total of 68% calves were ≤ 6 months of age, with a distribution of 62.5% (794/1271) of the total calves being female. Calf body weight had a median weight of 152 kg (range, 50–430 kg) across all individuals sampled Figure 1.

### FEC and larval differentiation

The median FEC across all farms was 100 epg (range, 0–6,700 epg) with each respective farm outlined in Table 1. A total of 38% (489/1271) animals recorded an FEC below the detectable limit. Mean FEC count for each treatment group on each respective farm is outlined in Table 2. The overall larval differentiation was 60% *Cooperia oncophora*, 25% *Ostertagia*, 8% other *Cooperia* species, 5% *Trichostrongylus* and 2% *Haemonchus*, respectively. Larval differentiation for each individual farm is outlined in Table 3.

**Table 1:** Faecal egg count (FEC) descriptive results in eggs per gram across 14 dairy farms sampled in the south west region of Western Australia between June and December 2020

Farm	Median FEC^a^	Minimum FEC	Maximum FEC
1	0	0	300
2	100	0	5,000
3	700	0	4,700
4	0	0	400
5	100	0	2,900
6	200	0	3,300
7	100	0	2,800
8	1,250	100	6,700
9	0	0	400
10	300	0	5,300
11	100	0	1,000
12	400	0	3,400
13	100	0	2,500
14	100	0	1,100

Values presented as zero were below the detectable range.

^a^ Median FEC was used as FEC failed to fit within a normal distribution.

**Table 2:** Mean faecal egg count in eggs per gram of strongyle species for each anthelmintic group tested on 11 dairy farms in the south west region of Western Australia between June – December 2020

Farm no.	Control	Doramectin	Levamisole/Abamectin	Levamisole	Fenbendazole
	Mean epg	Mean epg	Mean epg	Mean epg	Mean epg
2	82	70	46	4	124
3	666	453	297		
5	499^a^ / 242^b^	25^b^	10^a^	13^b^	288^a^
6	393	94	15	11	6
7	293	32	3	2	11
8	1,332	305	928	128	467
10	119	65	0	5	16
11	150	151	0	0	26
12	363	21	29	4	21
13	99	1	1	3	8
14	202	130	3	8	132

Two separate controls were used due to calf availability.

^a^ Levamisole/Abamectin, Fenbendazole.

^b^ Doramectin, Levamisole.

**Table 3:** Larval differentiation in percentage of strongyle species for 11 dairy farms in the south west region of Western Australia between June – December 2020

Farm no.	*Ostertagia*	*Trichostrongylus*	*Cooperia oncophora*	*Cooperia spp*.	*Oesophagostomum*	*Haemonchus*
	Larval %	Larval %	Larval %	Larval %	Larval %	Larval %
2	42	2	56			
3	16	1	68			15
5^a^	57	4	23	16		
5^b^	10		79	11		
6	6		92			2
7			90	10		
8	4	8	84	4		
10	10	2	81	7		
11	3		84	13		
12	6	1	87	6		
13	21	44	29	1	5	
14	7		70	22	1	

Larval differentiations are based of the control group in each farm. Two separate controls were used due to calf availability.

^a^ Levamisole/Abamectin, Fenbendazole.

^b^ Doramectin, Levamisole.

### Anthelmintic resistance

Anthelmintic resistance was strongest within the doramectin class 91% (10/11) of the farms, followed by the fenbendazole class in 80% (8/10) of the farms. Anthelmintic resistance was weakest in the levamisole class in 10% (1/10) of the farms, followed by the levamisole/abamectin combination in 31% (4/11) of farms. Average overall FECR was highest in levamisole with a reduction of 96%, followed by levamisole/abamectin combination of 83% overall reduction. The lowest overall FECR was present in doramectin with a 59% reduction, followed by an overall reduction of 64% in fenbendazole. A summary of anthelmintic resistance within each farm is outlined in Table 4.

**Table 4:** Percentage reductions in strongyle species for each anthelmintic group tested on 11 dairy farms in the south west region of Western Australia between June – December 2020

Farm no.	Doramectin	Levamisole/Abamectin	Levamisole	Fenbendazole
	FECR (%)	FECR (%)	FECR (%)	FECR (%)
2	**15**	**44**	95	−**50**
3	**32**	**55**		
5	**90**	98	95	**42**
6	**76**	96	97	98
7	**89**	99	99	96
8	**77**	**30**	**90**	**65**
10	**46**	100	96	**86**
11	**−1**	100	100	**83**
12	**94**	**92**	99	**94**
13	99	99	97	**92**
14	**35**	98	96	**35**

Reduction was calculated based on comparisons to the control group. Values in bold represent where resistance (<95% faecal egg count reduction (FECR)) was present.

*C. oncophora* showed strongest resistance against doramectin on 91% (10/11) of the farms and to fenbendazole on 64% (7/11) of the farms. *Ostertagia* showed strongest resistance to fenbendazole on 80% (8/10) of the farms and in 70% (7/10) of the farms to levamisole. *Trichostrongylus* showed resistance, in doramectin and levamisole/abamectin combination in 14% (1/7) of the farms. The proportion of resistance for each anthelmintic at a species level is outlined in Table 5.

**Table 5:** Proportion of properties with anthelmintic resistance (<95% faecal egg count reduction) at species level across 11 farms in the south west region of Western Australia between June – December 2020

Active ingredient	*Ostertagia*	*Trichostrongylus*	*Cooperia oncophora*	Cooperia spp.	At least one spp.
Doramectin (ML)	0/10 (0%)	1/7 (14%)	10/11 (91%)	7/8 (88%)	10/11 (91%)
Abamectin/ levamisole (ML/LV)	5/10 (50%)	1/7(14%)	4/11 (36%)	3/8 (38%)	6/11 (55%)
Levamisole (LV)	7/10 (70%)	0/7 (0%)	0/11 (0%)	0/8 (0%)	7/10 (70%)
Fenbendazole (BZ)	8/10 (80%)	0/7 (0%)	7/11 (64%)	6/8 (75%)	9/10 (90%)

### Questionnaire

A total of 12 out of 14 questionnaires were completed **(** 6, 7 **)**. All farms utilised rotational grazing. Of these, only 42% (5/12) treated their cattle with anthelmintics prior to moving their stock on to another paddock. A total of 92% (11/12) of the farms used anthelmintics. Of the farms which used anthelmintics, only 50% (6/12) utilised a quarantine drench when treating new stock. All farms reported using macrocyclic lactones or combination anthelmintics on their cattle.

**Table 6:** Herd characteristics and pasture management practices of dairy farms sampled in the south west region of Western Australia

Farm	Dairy Enterprise							Pasture management						
	Herd size	Calving pattern	Predominant breed	Grazing	BJD Strategy^a^	Introduced stock	Bio‐security	Rotational grazing	Rest period	Seasonal differ?	Rest period	Drench Period^d^	Cattle death due to GIN	Experienced anthelmintic resistance
1	700	Split	Holstein‐Friesians	Both	Yes	No	Yes	Yes	Yes	Yes	Season dependent	Prior	No	No
2	350	Split	Holstein‐Friesians	Dry	Yes	Not often	Yes	Yes	Yes	Yes		Prior	No	No
3	95	Year round	Aussie red	Dry	No	No	Yes	Yes	Yes	Yes	17–35 days	Variable	No	No
4	600	Year round	Crossbreed	Both		Yes^b^	Yes	Yes	Yes	Yes	Season dependent	Other	No	No
5	180	Year round	Holstein‐Friesians	Both	Yes	No	Yes	Yes	Yes	Yes	Season dependent		No	No
6	300	Year round	Holstein‐Friesians	Dry	Yes	Yes^b^	Yes	Yes	Yes	No			No	Yes
7	360	Year round	Holstein‐Friesians	Both	Yes	Sires^c^	Yes	Yes	Yes	Yes	3–6 weeks	Prior	No	No
8	570	Split	Crossbreed	Both	Yes	Sires^c^	No	Yes	Yes	Yes	Leaf emergence rate	Variable	Yes	No
10	3,200	Split	Crossbreed	Both	No	Sires^c^	No	Yes	Yes	Yes	20–40 days	Prior	Unsure	Unsure
11	150‐300	Spring	Holstein‐Friesians	Both	Yes	Yes^b^		Yes	Yes	Yes	Variable	Variable	Yes	No
12	550	Split	Holstein‐Friesians	Both	Yes	No	Yes	Yes	Yes	Yes		Prior	No	Unsure
13	830	Split	Crossbreed	Dry		No		Yes	Yes	No			Yes	No

^a^ Implement a Bovine Johne's Disease (BJD) strategy where calves are isolated from adult cattle for the first 12 months of age.

^b^ Yes: >10% of stock is introduced to the property.

^c^ Sires: bulls were only introduced cattle to the property.

^d^ Drenching period for cattle regarding moving pastures.

**Table 7:** Anthelmintic usage and worm control practices of dairy farms sampled in the south west region of Western Australia

Farm	Anthelmintic usage and worm control							
	Quarantine drench	Anthelmintic class	Estimate weight at treatment	Estimation method	Treated group	Annual anthelmintic treatment frequency		
						Weaners (0–12 months)	Heifers (12–24 months)	Milking Herd (>24 months)
1	Yes	ML	Yes	Herd average	Individual groups	2	2	1
2	No	ML	Yes	Guess individual weight	Individual groups	2	1	Individuals
3	No		Yes	Guess individual weight	Individual groups	2	1	1
4	Yes	ML	Yes	Overestimating	Individual groups	2	4	2
5	Yes	ML	No		Individual groups	1	1	1
6	Yes	ML	Yes	Guess individual weight	Select individuals	2	1	Individuals
7	Yes	ML	Yes	Guess individual weight	Individual groups	2	1	1
8	No	ML	Yes	Herd average	Individual groups	2–3	1–2	0
10	No		Yes	Overestimating		3	1	0
11	No	ML	Yes	Overestimating	Individual groups	2	1	0
12	Yes	Combination and ML	Yes	Heaviest cow's weight	Individual groups	4	3	0
13	This section was left blank as enterprise does not use anthelmintic treatment and relies on rotational grazing							

The methods utilised for estimating stock weight at treatment varied amongst farms. No farms reported using weighing scales to determine the weight. Of the farms, 66% (8/12) reported treating their weaners twice a year. One farm reported treating weaners with anthelmintics once per year, and the remaining two reported treating three and four times a year. Previous problems regarding anthelmintic resistance were reported from one farm. Anecdotal cattle death due to GIN burden was reported on 25% (3/12) of the farms.

## Discussion

The primary objective of the study was to determine the prevalence of GIN among weaned bull calves and replacement heifers between 4 and 12 months and to quantify the level of anthelmintic resistance of dairy farms within a pasture‐based production system. The results of this investigation clearly indicate a strong level of anthelmintic resistance, with at least one class of anthelmintic failing to achieve a 95% reduction in FEC in one or more GIN species per farm.

*C. oncophora* was the most prevalent species with significant anthelmintic resistance to doramectin. The finding of resistance to doramectin by injection in *C. oncophora* in 91% of the farms is consistent with prevalence figures reported in previous studies^10^, ^16^, ^25^ confirming predisposition for macrocyclic lactone resistance in this genus. The level of resistance in this survey is concerning as all farms reported use of macrocyclic lactones in the treatment of stock. The lack of production impact recognised by farmers could be attributed to the prevalence of *C. oncophora*, generally considered of low pathogenicity,^4^ though heavy worm burdens can result in clinical disease and significant production losses. A recent New Zealand report cited a live weight loss of 14 kg in calves at 12 months of age, which was attributed to resistant *C. oncophora*.^26^ In addition, a study examining the effects of experimental infections of macrocyclic lactones‐resistant *C. punctata* in steers reported a decrease in live weight gain of 7.5% (P = 0.02) and a reduction in dry matter intake of 680 grams per day (P = 0.02).^6^

Resistance to macrocyclic lactones in *Ostertagia* was not detected on any of the farms tested, although resistance has been detected in Victorian cattle.^10^, ^25^ Cotter *et al* (2015) reported a lower efficacy of macrocyclic lactones in *Ostertagia* through pour‐on formulations where 25% of the farms tested had <95% FECR at day 14, compared to the injectable formulation which was fully effective. Additionally, within *C. oncophora*, there was little difference between injectable and pour‐on at day 14, with mean reductions of 85% and 93%, respectively. Prevalence of resistance in *Ostertagia* towards macrocyclic lactones has not been reported in Western Australia. However, with the popularity of pour‐on formulations, there is a risk of resistance.

To the authors knowledge, this is the first report of fenbendazole resistance within *Cooperia* in Western Australian dairy cattle. Resistance to fenbendazole was common in this study with 80% of farms failing to achieve ≥95% FECR. Resistance was strongest in *Ostertagia* (80%) and *C. oncophora* (64%). However, it should be noted that at dosages used within this study, fenbendazole has an 80% efficacy against developing stages of *Ostertagia*.^27^ Previous resistance has been reported to *Ostertagia* and *C. concophora*.^10^, ^15^, ^25^ Unfortunately, there is little information regarding the management of anthelmintic resistance in cattle.^26^ A study in New Zealand beef cattle reported rare benzimidazole usage, occurring in combination with either levamisole or macrocyclic lactones.^13^ Similar results were reported in this study, where no farmers reported use of benzimidazoles in their properties. These results suggest that before further usage, fenbendazole efficacy at varying dosages should be tested within the property or used in combination with additional anthelmintics for broad‐spectrum coverage.

Levamisole remained highly effective against nematodes of cattle, with resistance only found within *Ostertagia* species in this study. However, reduced efficacy in *Ostertagia* could be attributed to the rapid replacement of adults by larval stages during treatment intervals where there was failure to remove inhibited and developing larvae.^3^, ^28^

The mixed nature of worm infection in all cattle herds and the contrasting efficacies of anthelmintics reported in this study creates challenges for effective worm control. Levamisole was highly effective in the control of *Cooperia* species, yet performed poorly against *Ostertagia*. Macrocyclic lactones, on the other hand, had high efficacy against *Ostertagia* but performed poorly to control *Cooperia*. A combination anthelmintic is likely to be most effective^16^ to both maintain animal health and keep resistant genes as scarce as possible.^11^ Adopting combination treatments prior to development of resistance is important to maintain their efficacy.^29^ Within Australia, there are currently several different registered combination anthelmintics for use in cattle, including Trifecta® (levamisole/abamectin/oxfendazole MSD Animal Health Australia), Eclipse® (levamisole/abamectin, Boehringer Ingelheim) and Cydectin Platinum® (moxidectin/levamisole, Virbac), but their use by farmers is very low, especially compared to New Zealand farmers.

Within this study, the levamisole/abamectin combination achieved a high level of FECR (≥95%) on 69% of the farms. However, on some farms, the FECR was less than levamisole alone. These results are significantly different to previous studies, where a levamisole/abamectin combination was fully effective against GIN.^25^, ^30^ Within this study, the levamisole/abamectin combination was the only topical method of treatment; therefore, decreased efficacy could be as a result of inaccurate doses of anthelmintics due to the effect of weather conditions on anthelmintic performance^31^ and potential licking behaviour.^32^ Furthermore, within a conventional environment, inaccurate dosages can also be attributed to farmers using indirect methods of estimating cattle weight instead of calibrated scales to determine the appropriate dosage.

Sustainable control of GIN requires additional strategies to solely anthelmintics. Refugia is a key asset in the sustainable control of GIN and viability of future anthelmintic treatments.^5^, ^33^ Pasture contaminated with susceptible GIN larvae from free‐living stages or untreated animals form a prime source of refugia, allowing for a decreased rate of resistance development providing there is not heavy anthelmintic reliance.^7^, ^12^, ^33^ The lack of anthelmintic usage and reliance on grazing management and refugia for GIN control was reported on one farm within this study, with resistance only evident in the fenbendazole group (FECR 92%). Reliance on refugia and grazing management is a key factor in minimising the development of resistance without compromising stock production.

The challenge exists in finding the optimal proportion of refugia to minimise anthelmintic resistance development, whilst maintaining animal performance. Two approaches are considered to optimise anthelmintic treatments^34^; targeted treatment (TT; whole groups treated after diagnostic information) and targeted selective treatment (TST; selected individuals treated within a group based on individuals diagnostic information). TST approaches have shown to be effective in sheep using various criteria for selection of individual treatment, including live weight or live weight gain.^35^, ^36^ The Happy Factor™ TST utilises individual animal weight predictions to determine required treatment, based on single animal failures to reach a predicted weight threshold.^37^ This method of TST has shown to slow the development of resistance,^38^, ^39^ where the standard threshold is transferable between farms, allowing for refinement using local data in cases where farm and animal specific characteristics are required.^37^ Other individual‐animal treatment decisions such as the FAMACHA© system for the clinical evaluation of anaemia due to haemonchosis in small ruminants have also proved feasible.^39^, ^40^ Attempts of implementing TST concepts for cattle have been made^41^, ^42^ with studies showing substantial reduction in anthelmintic treatments; however, small production losses have been associated to the TST.

It is recommended that farmers conduct regular FECRT to assess the efficacy of anthelmintics used on their farms; however, the test is seldom used as most producers have not perceived resistance on their farm and the expense of conducting FECRT is seen as uneconomical.^43^ Reducing the cost of FECRT may facilitate an increase in testing.^44^ Several studies have reported a high correlation and substantial level of agreement in FEC and FECR between individual and pooled sampling methods in both sheep^44^ and cattle,^43^ confirming the validity of pooled sampling. Furthermore, George et al reported a reduction in the number of samples to evaluate FEC or anthelmintic efficacy by 79.2%, significantly reducing the expense of testing. Therefore, pooled sampling can significantly reduce the cost and labour associated with FECRT.

## Conclusion

The results of the current study revealed anthelmintic resistance in the major species of cattle to all available anthelmintics is widespread in dairy farms within a pasture‐based production system of south west Australia. The current usage of anthelmintics available without regular resistance testing risks inadequate efficiency and further anthelmintic resistance development. Management strategies such as routine FECR testing and selective treatment is recommended to guide decision‐making of appropriate anthelmintics with adequate efficacy and optimal productivity on farm.

## Conflict of interest and sources of funding

The authors declare no conflicts of interest for the work presented here. This project was jointly funded by Western Dairy, Dairy Australia, Boehringer Ingelheim and Murdoch University.

## Supporting information


**Data S1.** Supporting Information.
