# Supplementary material for: Anthelmintic resistance of gastrointestinal nematodes in dairy calves within a pasture‐based production system of south West Western Australia
Source: Aust Vet J. 2022 Apr 5;100(7):283–91. doi: 10.1111/avj.13162 (PMC9542819; doi:10.1111/avj.13162)
Supplement: Supplementary file 1 — Data S1. Supporting Information. [file AVJ-100-283-s001.docx]

**Appendix A**: Survey template for determining farm details, pasture management and anthelmintic usage in tested farms.

Dairy enterprise

Milking herd size? (approx.): _________

Calving pattern? *Split/Year round/Spring/Other (Please specify) _______*

Pre-dominant breed in herd? *Holstein-Friesians/Crossbred/Other ________*

What form of grazing is used for the cattle on the property? *Dry/Irrigated/Both*

Does your farming enterprise implement a Bovine Johne’s Disease (BJD) control strategy on farm? (*Calves and young stock are isolated from adult animals until at least 12 months of age*) *Yes/No*

Do you introduce stock into your farming enterprise? *Yes (<10% of herd is introduced)/No/Sires/ Other____*

Do you have a farm biosecurity plan? *Yes/No*

Worming control practices

Do you drench your new cattle within two days of arrival on the property? (*Quarantine drench*) *Yes/No*

Regarding your quarantine drench: What drench do you currently use? *Please circle one or more: Combination/ML/Bezimidazole/Levamisole*

If possible, can you recall which specific drench product is used? ______________

When drenching, do you estimate the weight of your cattle? *Yes/No*

Which method do you use? *Overestimating/Herd average/Scales/Heaviest Cow’s Weight/Guess Individuals’ Weight/Other*

When drenching, which cattle do you drench? *All at once/Individual groups/Select Individuals/Other*

Please specify: _____________

What is your annual treatment frequency within your enterprise? *Weaners (0-12 Months) ______/Heifers (12-24 Months) ______/Milking Herd (>24 Months) _____*

Pasture management and drenching

Do you use rotational grazing? *Yes/No*

Does your pastures have a rest period? *Yes/No*

Does it differ seasonally? *Yes/No*

If so, how long? ___________

Which method(s) best describes your drenching period? *Prior to moving pasture/Immediately after moving pasture/ After having been on the pasture for an extended time/Variable/Other _____*

In the last 1 – 2 years, have any of your calves or cows died because of a worm burden/ problem? *Yes/No*

If yes, approximately how many calves __________ and cows_______________

In the last 1 – 2 years, have you experienced a drench resistance problem in any of your animal mobs? *Yes/No*

**Appendix B:** Strongyle faecal egg counts, larval differentiations and drench efficacy percentage at species level for 11 dairy farms which underwent anthelmintic resistance testing in the south west region of Western Australia between June – December 2020.

| Farm | Treatment | EPG (Mean) | Larval % | | | | | | Anthelmintic Efficacy % | | | | | | |
| --- | --- | --- | --- | --- | --- | --- | --- | --- | --- | --- | --- | --- | --- | --- | --- |
|  |  |  | Ostertagia | Trichostrongylus | Haemonchus | C. Oncophora | Cooperia spp. | Oesophagostomum | Overall | Ostertagia | Trichostrongylus | Haemonchus | C. Oncophora | Cooperia spp. | Oesophagostomum |
| 2 | Control | 82 | 42 | 2 |  | 56 |  |  |  |  |  |  |  |  |  |
|  | Doramectin | 70 |  |  |  | 100 |  |  | 15 | 100 | 100 |  | -52 |  |  |
|  | Levamisole/Abamectin | 46 | 11 |  |  | 89 |  |  | 44 | 85 | 100 |  | 11 |  |  |
|  | Levamisole | 4 | 100 |  |  |  |  |  | 95 | 89 | 100 |  | 100 |  |  |
|  | Fenbendazole | 124 | 24 |  |  | 76 |  |  | -50 | 14 | 100 |  | -104 |  |  |
| 3 | Control | 666 | 16 | 1 | 15 | 68 |  |  |  |  |  |  |  |  |  |
|  | Doramectin | 453 |  |  |  | 100 |  |  | 32 | 100 | 100 | 100 | 32 |  |  |
|  | Levamisole/Abamectin | 297 | 13 |  | 2 | 79 | 6 |  | 55 | 64 | 100 | 94 | 58 |  |  |
|  | Levamisole | Drenches not tested due to inadequate calf numbers | | | | | | | | | | | | | |
|  | Fenbendazole |  |  |  |  |  |  |  |  |  |  |  |  |  |  |
| 5 | Control 1 (ML-LV/BZ) * | 499 | 57 | 4 |  | 23 | 16 |  |  |  |  |  |  |  |  |
|  | Control 2 (ML/LV) * | 242 | 10 |  |  | 79 | 11 |  |  |  |  |  |  |  |  |
|  | Doramectin | 25 |  |  |  | 88 | 12 |  | 90 | 100 |  |  | 88 | 89 |  |
|  | Levamisole/Abamectin | 10 | 93 |  |  | 7 |  |  | 98 | 97 | 100 |  | 99 | 100 |  |
|  | Levamisole | 13 | 100 |  |  |  |  |  | 95 | 48 |  |  | 100 | 100 |  |
|  | Fenbendazole | 288 | 25 |  |  | 71 | 4 |  | 42 | 75 | 100 |  | -78 | 86 |  |
| 6 | Control | 393 | 6 |  | 2 | 92 |  |  |  |  |  |  |  |  |  |
|  | Doramectin | 94 |  |  | 2 | 98 |  |  | 76 | 100 |  | 76 | 75 |  |  |
|  | Levamisole/Abamectin | 15 | 28 |  | 6 | 66 |  |  | 96 | 82 |  | 88 | 97 |  |  |
|  | Levamisole | 11 | 64 |  |  | 35 |  |  | 97 | 71 |  | 100 | 99 |  |  |
|  | Fenbendazole | 6 | 22 |  | 53 | 25 |  |  | 98 | 94 |  | 60 | 100 |  |  |
| 7 | Control | 293 |  |  |  | 90 | 10 |  |  |  |  |  |  |  |  |
|  | Doramectin | 32 |  |  |  | 81 | 19 |  | 89 |  |  |  | 90 | 79 |  |
|  | Levamisole/Abamectin | 3 |  |  |  | 36 | 64 |  | 99 |  |  |  | 100 | 94 |  |
|  | Levamisole | 2 |  |  |  | 67 | 33 |  | 99 |  |  |  | 99 | 98 |  |
|  | Fenbendazole | 11 | 2 |  |  | 89 | 9 |  | 96 |  |  |  | 96 | 97 |  |
| 8 | Control | 1332 | 4 | 8 |  | 84 | 4 |  |  |  |  |  |  |  |  |
|  | Doramectin | 305 |  | 4 |  | 92 | 4 |  | 77 | 100 | 89 |  | 75 | 77 |  |
|  | Levamisole/Abamectin | 928 | 5 | 2 |  | 81 | 12 |  | 30 | 13 | 83 |  | 33 | -109 |  |
|  | Levamisole | 128 | 100 |  |  |  |  |  | 90 | -139 | 100 |  | 100 | 100 |  |
|  | Fenbendazole | 467 | 28 |  |  | 64 | 8 |  | 65 | -146 | 100 |  | 73 | 30 |  |
| 10 | Control | 119 | 10 | 2 |  | 81 | 7 |  |  |  |  |  |  |  |  |
|  | Doramectin | 65 |  |  |  | 81 | 19 |  | 46 | 100 | 100 |  | 46 | -48 |  |
|  | Levamisole/Abamectin | 0 | 36 |  |  | 36 | 28 |  | 100 | 100 | 100 |  | 100 | 100 |  |
|  | Levamisole | 5 | 100 |  |  |  |  |  | 96 | 58 | 100 |  | 100 | 100 |  |
|  | Fenbendazole | 16 | 18 |  |  | 56 | 26 |  | 86 | 76 | 100 |  | 91 | 49 |  |
| 11 | Control | 150 | 3 |  |  | 84 | 13 |  |  |  |  |  |  |  |  |
|  | Doramectin | 151 |  |  |  | 92 | 8 |  | -1 | 100 |  |  | -10 | 38 |  |
|  | Levamisole/Abamectin | 0 |  |  |  |  |  |  | 100 | 100 |  |  | 100 | 100 |  |
|  | Levamisole | 0 | 80 |  |  | 20 |  |  | 100 | 100 |  |  | 100 | 100 |  |
|  | Fenbendazole | 26 | 6 |  |  | 84 | 10 |  |  | 65 |  |  | 83 | 87 |  |
| 12 | Control | 363 | 6 | 1 |  | 87 | 6 |  |  |  |  |  |  |  |  |
|  | Doramectin | 21 |  |  |  | 83 | 17 |  | 94 | 100 | 100 |  | 94 | 84 |  |
|  | Levamisole/Abamectin | 29 | 8 |  |  | 67 | 25 |  | 92 | 90 | 100 |  | 94 | 67 |  |
|  | Levamisole | 4 | 100 |  |  |  |  |  | 99 | 84 | 100 |  | 100 | 100 |  |
|  | Fenbendazole | 21 | 32 |  |  | 52 | 16 |  | 94 | 69 | 100 |  | 97 | 84 |  |
| 13 | Control | 99 | 21 | 44 |  | 29 | 1 | 5 |  |  |  |  |  |  |  |
|  | Doramectin | 1 | 15 | 62 |  | 12 |  | 12 | 99 | 99 | 98 |  | 99 | 100 | 97 |
|  | Levamisole/Abamectin | 1 |  | 60 |  | 40 |  |  | 99 | 100 | 98 |  | 98 | 100 | 100 |
|  | Levamisole | 3 | 43 | 57 |  |  |  |  | 97 | 95 | 97 |  | 100 | 100 | 100 |
|  | Fenbendazole | 8 | 9 | 2 |  | 88 |  | 1 | 92 | 97 | 100 |  | 77 | 100 | 98 |
| 14 | Control | 202 | 7 |  |  | 70 | 22 | 1 |  |  |  |  |  |  |  |
|  | Doramectin | 130 |  |  |  | 95 | 5 |  | 35 | 100 |  |  | 12 | 85 | 100 |
|  | Levamisole/Abamectin | 3 |  |  |  | 100 |  |  | 98 | 100 |  |  | 98 | 100 | 100 |
|  | Levamisole | 8 | 83 |  |  | 14 | 3 |  | 96 | 51 |  |  | 99 | 99 | 100 |
|  | Fenbendazole | 132 | 8 |  |  | 81 | 11 |  | 35 | 25 |  |  | 24 | 67 | 100 |

*Two controls were used as two separate visits were conducted to facilitate calf numbers required. Control 1 (Levamisole/Abamectin and Fenbendazole), Control 2 (Doramectin and Levamisole)
